# A programmable hierarchical-responsive nanoCRISPR elicits robust activation of endogenous target to treat cancer

**DOI:** 10.7150/thno.62449

**Published:** 2021-10-11

**Authors:** Chao Liu, Ning Wang, Rui Luo, Lu Li, Wen Yang, Xiye Wang, Meiling Shen, Qinjie Wu, Changyang Gong

**Affiliations:** State Key Laboratory of Biotherapy and Cancer Center, West China Hospital Sichuan University, Chengdu, 610041, P. R. China.

**Keywords:** programmable hierarchical-responsive, CRISPR/dCas9, transcriptional activation, cancer therapy, gene delivery

## Abstract

Despite promising progress of cancer gene therapy made, these therapeutics were still limited by the diversity of gene sizes and types. CRISPR/dCas9 mediated activation of tumor endogenous gene has shown great potential to surmount hinders of genetic varieties during the process of cancer gene therapy. However, the blood interference along with complicated tumor extra/intracellular microenvironment substantially compromise the performance of CRISPR/dCas9-based therapeutics *in vivo*.

**Methods:** In this study, we constructed a programmable hierarchical-responsive nanoCRISPR (PICASSO) that can achieve sequential responses to the multiple physiological barriers *in vivo*. The core-shell structure endows PICASSO with long blood circulation capacity and tumor target accumulation as well as efficient cellular uptake and lysosomal escape, leading to high-performance of CRISPR/dCas9-mediated gene activation, which favors the antitumor efficacy.

**Results:** Owing to these properties, PICASSO facilitated CRISPR/dCas9 mediated efficient transcriptional activation of various types of endogenous gene, and long non-protein-coding genes (LncRNA) containing targets ranging in size from ~1 kb to ~2000 kb in tumor cells. Intravenous administration of PICASSO to the tumor-bearing mice can achieve effective transcriptional activation of therapeutic endogenous gene, resulting in remarkable CRISPR/dCas9-mediate tumor inhibition with minimal adverse effect.

**Conclusions:** Taken together, these characteristics allow PICASSO to unleash the potential of CRISPR/dCas9-based therapeutics in oncological treatment. The study provides a simple and versatile strategy to break through the restriction of sizes and types against cancer by utilization of tumor endogenous gene.

## Introduction

Gene therapy, a form of treating disease by correcting the genetic instruction in patients' cells, have emerged as promising approaches for cancer treatment 1. To date, cancer gene therapeutics mainly including transgenic therapies and gene editing-based therapies 2-4. Current transgenic therapies were primarily depended upon cDNA overexpression, which limited by the large size of coding sequences, lack of isoform diversity and high cost of construction 5,6. On the other hand, despite the fact that encouraging progress of CRISPR/Cas9 technology were achieved, safety concerns, especially irreversible breaks induced in the human genome by gene editing increased the potential carcinogenic risk in clinical applications 7,8.

Recently, CRISPR/dCas9 based transcriptional activation system (CRISPRa), derived from CRISPR/Cas9 system, has emerged as a powerful tool for robust activation of endogenous gene expression 9,10. Distinct from double strand breaks of DNA caused by CRISPR/Cas9, Cas9 protein of CRISPRa system are repurposed into catalytically inactive form (dCas9) and fused to transcriptional activation domains, which possess the new ability to activate endogenous gene expression and avert introducing undesired permanent mutations in the genome 11,12. CRISPRa simply requires the sgRNA guidance to overcome abovementioned limitations, revolutionizing the way in cancer gene therapy. Taking advantage of the characteristics, CRISPRa system has provided a precise and natural way against tumors.

Nevertheless, the arrival of the CRISPRa system into target cells is the prerequisite to fully exert its function 13. Especially for cancer therapeutics *in vivo*, the CRISPRa system has to undergo multiple biological barriers before entry nucleus, encompassing interference of blood components, cytomembrane blockade and lysosomal degradation 14,15. Therefore, CRISPRa system can hardly surmount these obstacles without the assistance of delivery vehicle. To date, viral vectors including adenovirus, lentivirus, and adeno-associated virus, etc., are the mainly platforms for CRISPRa applications 16-18. However, limited packing capacity makes the preclinical study inevitable to rely on CRISPRa transgenic technology, which were not applicable for human therapy. Furthermore, immunogenicity and carcinogenesis of virus impede its clinical applications as well 19. Currently, safety concerns by the clinical applications have made non-viral approaches the promising therapeutic candidates 20-22. To achieve efficient delivery of CRISPR system, semiconductor polymer brush, DNA nanoclew and black phosphorus Nanosheets etc., have been developed 23-27. Although these studies displayed promising results, all the approaches have been designed and explored for CRISPR/Cas9 mediated genome editing 28-31. Inducing cell phenotypic changes that achieve antitumor therapeutic effects *in vivo* via CRISPR-based transcription activation required more optimized intratumor delivery efficiency than the standard genome editing which are remains elusive. Thus, a pragmatic strategy is needed to overcome these hurdles to ensure the full potential of CRISPRa system in therapeutic applications.

**Scheme 1:**
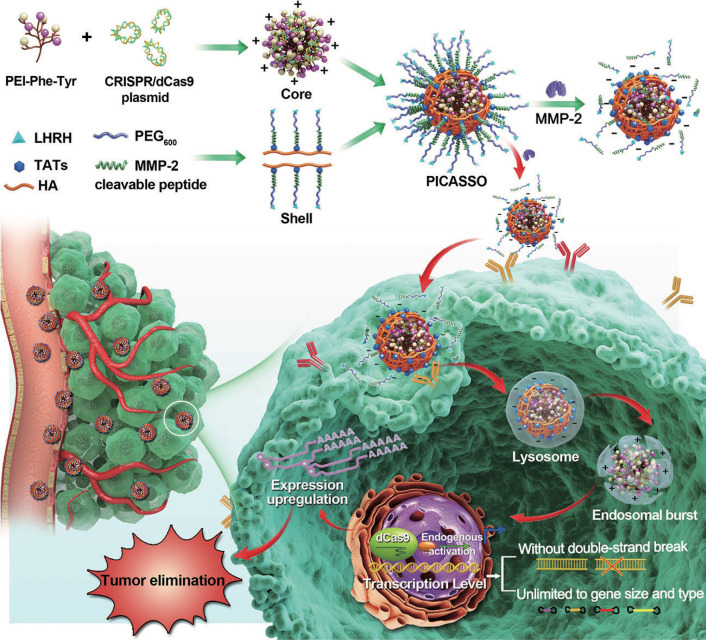
Schematic illustration for the nanoparticle fabrication and therapeutic workflow. The PICASSO is self-assembled from the multifunctional shell and CRISPRa polyplex electrostatic interaction and demonstrated on demand responses to the sequential physiological barriers *in vivo,* leading to efficient CRISPR/dCas9-mediated endogenous transcriptional activation without introducing genome changes and exogenous gene. This ability breaks the restrictions of gene size and type in genetic-based therapeutics and opens new avenue for oncological treatment.

For *in vivo* delivery, CRISPRa system needs to conquer sequential biological hurdles, including blood interference, cytomembrane obstruction and lysosomal degradation to realize its therapeutic potency 32. In light of these considerations, we constructed a programmable hierarchical-responsive nanoCRISPR (PICASSO) to overcome the biological hurdles in the order that more ideal for the antitumor therapeutics via CRISPR/dCas9-mediated transcriptional activation (**Scheme 1**). According to the designed concept, the PICASSO could exhibit programmed hierarchical-responsive properties after intravenous administration. Stage I: the PEGylated shell could improve the stability in the blood long circulation 33 whereas the luteinizing hormone-releasing hormone (LHRH) peptide and hyaluronic acid (HA) backbone endows PICASSO with dual actively targeting capability of tumors 34,35. Stage II: the “smart” matrix metalloproteinases-2 (MMP-2) responsiveness facilitate tumor penetration and cell uptake in the tumor microenvironment via dissociation of the PEG layer and exposure of cell penetrating peptide (HIV transactivator of transcription, TAT) 36. Stage III: After being internalized into tumor cells, the cationic polyplex could protect the CRISPRa cargos from the endo/lysosome capture and clearance by means of proton sponge effect. Finally, the CRISPRa system was released in the nucleus and highly efficient activated the expression of endogenous therapeutic gene at the transcriptional level without causing DNA double-strand breaks. During the whole process, PICASSO led to violent CRISPRa-mediated apoptotic phenotype of tumor cells and dramatically inhibition of tumor growth in xenograft models. Due to the advanced characteristics, the PICASSO platform could break the restrictions of gene size and types without genome changes, providing a new insight for cancer gene therapy and medical translation.

## Materials and Methods

### Materials

Sodium HA (*M_w_* of 35 kDa) was purchased from Freda Biochem. PEI with different molecular weight were obtained from Alfa Aesar (USA). The tumor-associated peptides: NH_2_-TATs-SH (CYGRKKRRQRRR) and LHRH-COOH (Ac-QHWSYk(c)LRP) were performed by Chinapeptides Co., Ltd. (China). NH_2_-PEG_600_-maleimide was by JenKem Technology Co., Ltd. (China). The MMP-2 enzyme (MMP-2) was purchased from PeproTech (USA). 3-(4,5-dimethylthiazol-2-yl)-2,5-diphenyltetrazolium bromide (MTT) was obtained from Sigma (USA). Proliferation assay EdU label kits were purchased from RIBOBIO CO., Ltd (China). Lipo 3000, LysoTracker Red, TOTO-3, Hochest 33342 and YOYO-1 were provided by Invitrogen (USA). The antibodies which were used for Western Blotting: mouse polyclonal anti-β-actin, rabbit polyclonal anti-TRAIL, rabbit polyclonal anti-Cleaved Caspase 3 and rabbit polyclonal anti-Cleaved Caspase 8 antibodies (Abcam, USA). Horseradish peroxidase (HRP)-labeled goat anti-mouse/rabbit secondary antibodies were purchased from CST (USA). TUNEL BrightRed apoptosis detection kits were acquired from Vazyme Biotech Co., Ltd (China).

### Cell culture and animals

Human HeLa cervical carcinoma cells and HEK-293T embryonic kidney cells were obtained from the American Type Culture Collection (ATCC; USA). Dulbecco's modified Eagle's medium (DMEM) medium contains 10% fetal bovine serum was purchased from Thermo Fisher Scientific (USA). The cells were incubated at 37 °C with a humidified atmosphere containing 5% CO_2_. All the cells were conducted by Hoechst DNA stain and agar culture methods fo*r Mycoplasma*-free detection.

Balb/c nude mice (6-8 weeks) were used for* in vivo* cervical carcinoma cancer model which were ordered from HFK Bioscience Co., Ltd. (China). All animal studies were approved by the Ethics Review Committee of Animal Experimentation of Sichuan University (Chengdu, P.R. China).

### Synthesis and characterization of core and shell

PPT was synthesized via amidation of PEI (1.8K) with Phenylalanine (Phe) and Tyrosine (Tyr). Briefly, Boc-Phe-COOH (25 mg), Boc-Tyr-COOH (25 mg), EDCI (25 mg) and NHS (20 mg) were dissolved in 1.5 mL N, N-dimethylformamide and stirred for 4 h. Subsequently, the Boc-Phe-CONHS and Boc-Tyr-CONHS were dropped into PEI 1.8K (100 mg) against DMSO. After stirred for 72 h, the mixture was dissolved in trifluoroacetic acid (TFA) for 6 h to detach the Boc. The TFA was removed by rotary evaporation at 40 °C for 10 min. The mixture was dialyzed against DMSO for 24 h, and then dialyzed against distill water for another 48 h. The grating ratios of Phe and Tyr on PEI 1.8K were confirmed by Ninhydrin assay according to our previous research 30, and calculated through the formula: X = (16 - 1.8 × C) / (0.196 × C + 1), where X is the number of fluorinated groups on PEI 1.8K, and C is the concentration of primary amine groups, mmol/L.

The synthetic route of shell was displayed (Figure S1). Firstly, 56.6 mg LHRH-COOH, EDCI (5 mg) and NHS (3 mg) were dissolved in 5 mL 2-(N-morpholino) ethanesulfonic acid (MES) buffer (pH 6.0) for 3 h to activate the -COOH on LHRH. Then, the NH_2_-PEG_600_-maleimide was added to the LHRH-CONHS solution and stirred for 48 h and dialyzed. Afterwards, the production was lyophilized for ^1^*H* NMR and FTIR. Secondly, LHRH-PEG_600_-maleimide and SH-MMP-2 substrate-TATs-NH_2_ were added in PBS for dissolution. After stirring for 48 h, the mixture was dialyzed against ddH_2_O. Thirdly, after HA (42.3 mg) with EDCI (2.3 mg) and NHS (1.4 mg) for 6 h, the HA-CONHS were added into the above mixture and stirred for another 48 h. After dialyzed with deionized water for 72 h, the shell was obtained followed by a lyophilization technique. Subsequently, the white powder was identified by ^1^*H* NMR and FTIR.

### Synthesis and characterization of the PICASSO

PICASSO was formed using an adsorption of positive and negative charges. Briefly, PPT with different mass ratio (0.5:1, 1:1, 2:1, 5:1, 10:1, 15:1) of pDNA in 100 μL PBS were incubated for 20 min, and then separated by 0.5% (w/v) agarose gel (Invitrogen, USA) in TAE (Tris-acetate-EMTA) buffer for 35 min. The gels were observed by the Gel Doc System (Bio-Rad) for determining the pDNA condense ability of PPT. We chose the different core@pDNA for cell transfection experiment. The preliminary tests showed that the transfection efficiency was significantly improved at the mass ratio of 10:1 (core@pDNA) which was used for further use. Core@pDNA was incubated with the shell (20:10:1) for another 30 min to form PICASSO. The pDNA condense ability of PICASSO was also determined by agarose gel.

The particle size and zeta potential of core@pDNA and PICASSO, as well as PICASSO + MMP-2 (pre-treated with 5 μg/mL MMP-2 enzyme for 1 h) were measured by DLS with a Malvern Nano ZS (Malvern, U.K.). The particle morphology was analyzed by TEM (JEOL JEM-100CX, Japan).

### Cell cytotoxicity analysis *in vitro*

MTT assay was performed to detect the viability of HeLa and 293T cells. The cells were seeded in 96-well plate (3,000 cells per well) for 24 h. The PEI 1.8K, PEI 25K, PPT, LHRH and the shell with set concentrations (0~200 μg/mL) were exposed to the cells. After incubated for 48 h, 5 mg/mL MTT was added for another 4 h. After the media were removed, the purple formazan crystals were dissolved by the addition of 150 μL DMSO (Thermo Fisher, USA). Finally, the media was measured at 570 nm by a microplate reader (Bio-Rad 680, USA). The cell viability was determined as a relative value (%) compared with the control, untreated cells (n = 6).

### Cellular uptake and intracellular trafficking of PICASSO

Primarily, the HeLa cells were labelled with anti-APC-CD44 antibody (Biolegend, USA) for 30 min at 4 °C, and then were analyzed by flow cytometry (BD) for the detection of CD44 receptors on HeLa. The cellular uptake of PEI 1.8K, PEI 25K, PPT, NON and PICASSO was measured by flow cytometry. Briefly, 1 × 10^5^ cells were seeded into 6-well plate. Subsequently, the pEGFP plasmid (2 μg/mL) was labeled with a YOYO-1 dye (Invitrogen, USA) which was at 1:5000 for 2 h. Then, the pEGFR/YOYO-1 was mixed with the PEI 1.8K (10:1), PEI 25K (1.33:1), PPT (10:1), NON (20:10:1) and PICASSO (20:10:1) for another 1 h. After that, the HeLa cells were incubated with different agents for 2 h and then were assembled for the measurement of green fluorescent signals and MFI by flow cytometry analysis (BD). As well, for the evaluation of MMP-2 enzyme sensitivity of PICASSO, the cellular uptake of NON +MMP-2 (5 μg/mL MMP-2 enzyme pre-treated for 1 h) and PICASSO + MMP-2 (5 μg/mL) was also detected by the above methods (n = 3).

The HeLa cells were seeded in confocal petri dishes for 12 h at a density of 2 × 10^5^ cells per well. YOYO-1 was labelled the plasmid (2 μg/mL) for 1 h and then mix with various transfection materials. The cells were incubated with PEI 25K@pDNA/YOYO-1, PICASSO/YOYO-1 and PICASSO/YOYO-1 + MMP-2 for another 0.5 h, 2 h and 4 h. At each time point, the lysosome was labelled with LysoTracker Red (1:5000) and the cell nucleus was stained with Hoechst 33342 (1:100) for another 10 min. The intracellular trafficking of PEI 25K, PICASSO and PICASSO + MMP-2 were observed by confocal fluorescence microscope system (ZEISS, LSM880, Germany) (n = 3).

### Transfection ability of PICASSO

For the transfection assay, 4 × 10^5^ HeLa cells were seeded into 6-well plate. After the DMEM growth medium (containing 10% FBS) was replaced, the cells were incubated with PEI 1.8K@pEGFP (10:1), PEI 25K@pEGFP (1.33:1), core@pEGFP (10:1), NON (20:10:1) and PICASSO (20:10:1) in fresh serum-free DMEM medium. Following incubation for 6 h, the medium was replaced and incubated for another 24 h. The images were taken by fluorescence microscope (100 ×) and the fluorescence signals in cells was analyzed using flow cytometry (BD) and quantified with FlowJo software (n = 3).

The cells were plated in the 6-well plate as mentioned above. After the medium was replaced with DMEM medium (containing 0%, 5%, 10%, 20% FBS), the cells were incubated with Lipo 3000@pEGFP, core@pEGFP (10:1), PICASSO (20:10:1), PICASSO + MMP-2 (20:10:1) for 6 h. Afterward, the culture media were replaced with fresh media (10% FBS) at 37 °C. The cells were collected after washing with PBS a density of 1 × 10^5^ cells, and then analyzed by flow cytometry (BD). The results were analyzed by FlowJo software (n = 3).

### Construction of CRISPRa system

The vectors used were based on SP-dCas9-VPR (from addgene) and PX330. The CRISPRa were cloned according to the Gibson assembly protocol. The final CRISPRa constructs harboring a sgRNA-TRAIL expression element and dCas9-VPR cassette. Sequence for Guide RNAs are provided (Table S1). Guide RNAs for activation of endogenous TRAIL are screened range from -1 to -2000 bp upstream of the transcription start site.

### Apoptosis and proliferation analysis

2 × 10^5^ HeLa cells were seeded into the 6-well plate for 24 h. After incubating with PBS, core@CRISPRa-Scramble, PEI 25K@CRISPRa-TRAIL, core@CRISPRa-TRAIL, NON, PICASSO, NON + MMP-2 and PICASSO + MMP-2, the cells were washed and centrifuged at 2000 rpm/min for 5 min to remove the waste medium. The apoptosis of HeLa cells was determined by Annexin V-FITC/PI Apoptosis Detection Kit (KeyGen, China) (n = 3). Briefly, the cells were stained with Annexin V-FITC for 15 min, and PI dye was incubated with cells for another 5 min. The apoptosis of cells was analyzed using flow cytometry (BD). All FITC^high^ and PI^high^ apoptotic cells were counted by FlowJo software. The proliferation assay was conducted using a EdU label kit according to the manufacturer's protocol. Briefly, the cells were incubated with the EdU solution and proceeded immediately to fixation and permeabilizayion steps. Subsequently, the sample was proceeded to fluorescent EdU detection by microscopic imaging.

### Western blot analysis

After being treated with PBS, core@CRISPRa-Scramble, PEI 25K@CRISPRa-TRAIL, core@CRISPRa-TRAIL, NON, PICASSO, NON + MMP-2 and PICASSO + MMP-2, the cells were washed with cold PBS, and then were lysed in ice-cold RIPA buffer (Cell Signaling Technology, CST, USA) with 1% cocktail (Roche, USA) according to the manufacturer^'^s introductions. The protein of different samples was extracted into SDS buffer (Bio-Rad, USA). Subsequently, the protein samples were separated via SDS-PAGE and transferred to a PVDF membrane (Millipore, USA). The membrane was incubated with primary antibodies: mouse anti-β-actin, rabbit anti-Cleaved Caspase 3 and rabbit anti-Cleaved Caspase 9 (CST, USA). All the primary antibodies were diluted in 1:1000. Following incubation for 20 h, the membrane was incubated with secondary antibodies (BD, USA) (1:10000) for another 1 h at 37 °C. After washing for three times with PBST, immunodetection of the proteins on the membrane was observed by the chemiluminesce system (Millipore, USA).

### *In vivo* distribution

Xenograft tumor model was established by injection of HeLa cells in the right flank of Balb/c nude mice (6-8 weeks old). When the tumor grown to 400 mm^3^, the mice were grouped randomly for two groups (n = 6). The plasmids were labelled with TOTO-3 dye (1:5000, Thermo Fisher) which was the high-sensitivity probes for nucleic acid detection and was a carbocanine dimmer with far-red fluorescence (647 nm). Each mouse was administrated with NON (20:10:1, 5 mg of pDNA per mouse) and PICASSO (20:10:1). Following administrated for 2 h, 4 h, 8 h, 12 h and 24 h, the mice were pictured* in vivo*, and then sacrificed for *ex vivo* visualization by IVIS Lumina Living Imaging system (Caliper, USA). The mean fluorescence intensity was also calculated.

### Tumor model establishment and *in vivo* anti-tumor evaluation

The HeLa cells (1 × 10^6^ cells per mouse) were implanted in the right flank of BALB/c nude mice (6-8 weeks old). When the tumor reached to ~100 mm^3^, the mice were randomly divided into five groups (n = 6) with the intravenous treatment of PBS, naked plasmid of CRISPRa (5 mg per mice), the shell of PICASSO, NON (20:10:1, 5 mg of CRISPRa-TRAIL plasmid per mouse) and PICASSO (20:10:1, 5 mg of CRISPRa-TRAIL plasmid per mouse) formulations once every 3 days. Tumor volume and body weight were recorded once every 2 days. The tumor length (L, mm), width (W, mm) and body weight were measured every other day. The tumor volume (V) was calculated using the equation: V (mm^3^) = L × W^2^ × 0.5. The mice were sacrificed at day 24. The harvested tumors and major organs were fixed in 4% neutral buffered paraformaldehyde (PFA) for IHC analysis and H&E staining.

### Histology and immunohistochemistry

The above tumors tissues and major organs were embedded in paraffin and sliced into a thickness of 6-8 μm for H&E histopathological staining.

The tumor tissue slides of different groups were performed immunohistochemical studies. The sections were dewaxed and rehydrated with xylene, gradient ethanol (100%, 90%, 75% concentration) and deionized water for different times followed the instructions. The endogenous peroxidase was removed with hydrogen peroxide for 30 min. After blocking with 10% normal goat serum for 1-2 h, the slides were stained with 100 μL mouse anti-human TRAIL antibody (1:100) and rabbit anti-human Ki67 (1:100) antibody, respectively. After incubating overnight at 4 ^o^C, excess primary antibodies were removed and 100 μL secondary antibody was incubated for another 1 h. The brown color was visualized, and the images were captured by microscope (200 ×). The brown color on the slides were counted by dividing the brown area by the total area in six random fields using Image J for calculation of TRAIL index and Ki67 labelling index (Ki 67 LI).

TUNEL assay was investigated in per group. Briefly, the tumor slides per group were subjected to TUNEL staining using a TUNEL BrightRed Apoptosis Detection Kit (BD). The apoptotic cells were distinguished by red, and the nuclei were identified by Hoechst 33342 staining (blue). The pictures were taken by fluorescence microscope (400 ×). The apoptosis index was computed in four random equal-size fields of the slide.

### Statistical analysis

GraphPad Prism 7 and SPSS were used for analyzing statistical significance. The *t*-tests were utilized for comparison between two groups. The multiple groups were analyzed utilizing a one-way analysis of variance (ANOVA) followed by the appropriate post-hoc tests. The threshold which was considered statistically significance was p < 0.05.

## Results and Discussion

### Synthesis and characterization of PICASSO

The PICASSO nanocomplex was composed of two parts, the cationic core and multifunctional shell. Firstly, the amino acid modified polyethyleneimine (PPT, the core of PICASSO) was synthesized by modifying polyethyleneimine 1.8 kDa (PEI 1.8K) with phenylalanine (Phe) and tyrosine (Tyr). Then the chemical structures were determined by ^1^*H* nuclear magnetic resonance analysis (^1^*H* NMR) (Figure S2). According to the ninhydrin analysis, the average number of Phe and Tyr modified on PEI 1.8K was confirmed. PPT35 (the grafting ratio of Phe and Tyr was 35%) had an excellent transfection efficiency in the preliminary tests, so PPT35 was chosen to construct the polyplex (core@CRISPRa). Secondly, the multifunctional shell of PICASSO (LHRH polypeptide ligands-PEG_600_-MMP-2-cleavable linker-TATs-HA) was synthesized. Subsequently, the PICASSO was obtained by coating the shell on core@CRISPRa polyplex through electrostatic interaction. For comparation, the MMP-2-nonresponsive shell was synthesized and self-assembled into nanocomplex to afford MMP-2-nonresponsive nanoparticles (NON).

**Figure 1:**
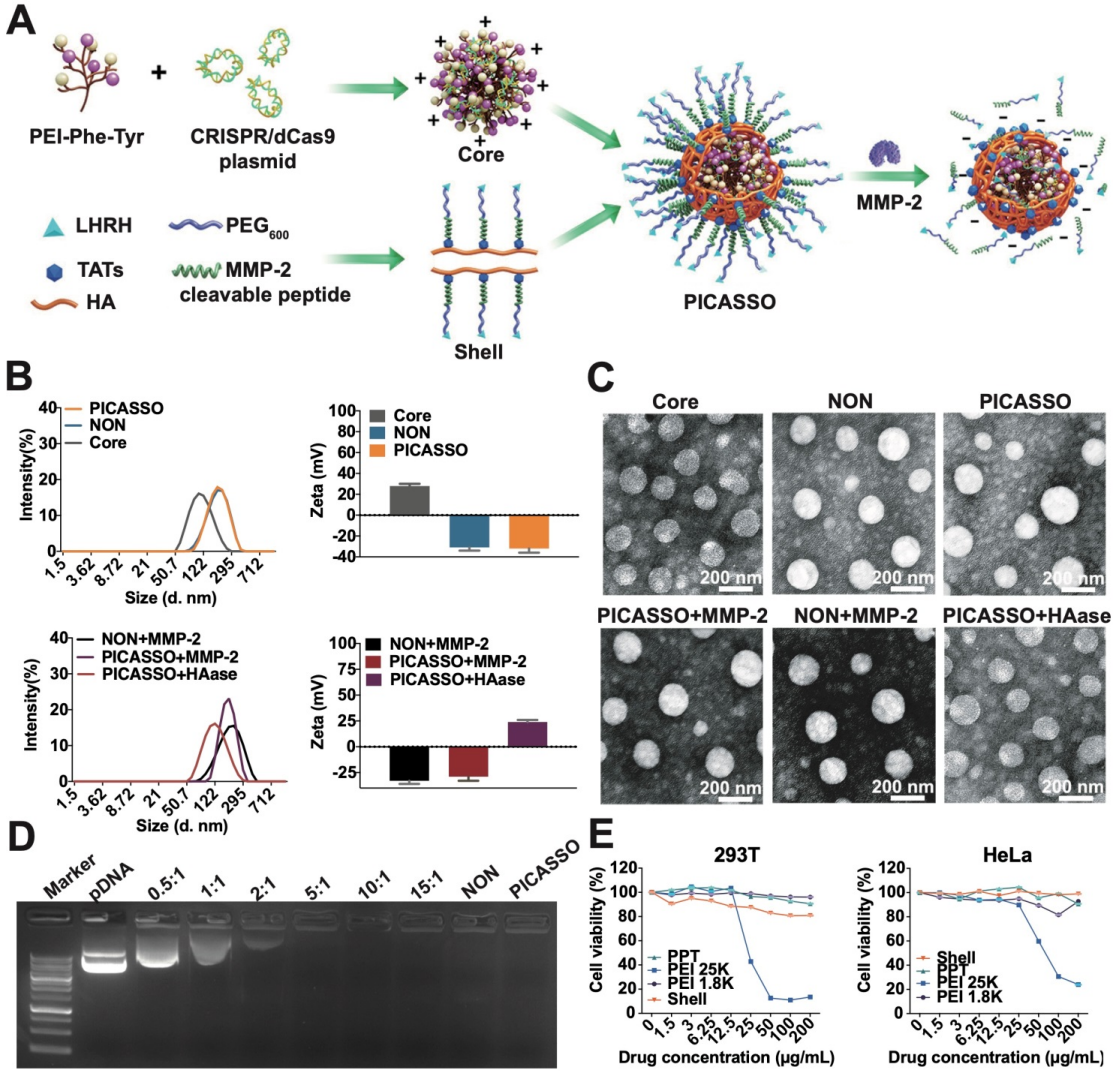
** The characterization of PICASSO.** (**A**) Representative the preparation of PICASSO. (**B**) Size distribution and surface charge. (**C**) TEM images of PICASSO in simulated tumor conditions. (**D**) Plasmid condensation assay. Lane 1-9: DNA marker, CRISRPa plasmid (10 kb), PPT35@plasmid polyplexes at different polymer to plasmid weight ratios (0.5:1, 1:1, 2:1, 5:1, 10:1, 15:1), NON and PICASSO (shell:core:plasmid, 25:10:1). (**E**) Cytotoxicity of different groups by MTT assay.

Dynamic light scattering (DLS) measurement and transmission electron microscopic (TEM) revealed that core@CRISPRa polyplex or PICASSO showed spherical morphology and hydro-dynamic size was 100.2 ± 4.2 nm or 145.3 ± 2.1 nm, with zeta potential of +28.1 ± 0.4 mV or -34.2 ± 0.7 mV respectively (Figure 1A-C). Next, the MMP-2 and HA enzyme was utilized to simulate the enzyme rich microenvironment of extra/intratumor. The particle size of PICASSO pre-incubated with MMP-2 decreased from 145.3 ± 2.1 nm to 122.3 ± 0.6 nm. The surface charge switched from -34.2 ± 0.7 mV to -36.2 ± 0.4 mV. Meanwhile, due to the hyaluronidase-mediated digestion of HA backbone from the PICASSO, the diameter decreased from 145.3 ± 2.1 nm to 110.1 ± 2.2 nm, and the zeta potential switched from -34.2 ± 0.7 mV to +25.1 ± 0.5 mV. The above data indicated that the PICASSO had homogeneous morphology and agile enzyme responsive characteristics in the simulated microenvironment. Afterward, we explored the plasmid condensation capacities of PPT35, NON and PICASSO. As shown in Figure 1D, the PICASSO could retard plasmid mobility effectively.

Furthermore, to determine the impact of nanocomplex on the cell viability, we first evaluated the cytotoxicity in HEK-293T and HeLa cells (Figure 1E). Branched polyethyleneimine 25 kDa (PEI 25K), a golden standard of non-viral gene delivery system, was used as a control. PEI 1.8K showed minimal toxicity in both cells. The core and the shell of PICASSO showed lower toxicity than PEI 25K. Even at 200 μg/mL, no obvious cytotoxicity was observed. These results demonstrated that PICASSO was an ideal carrier for *in vivo* application.

### Cell internalization and intracellular trafficking

**Figure 2:**
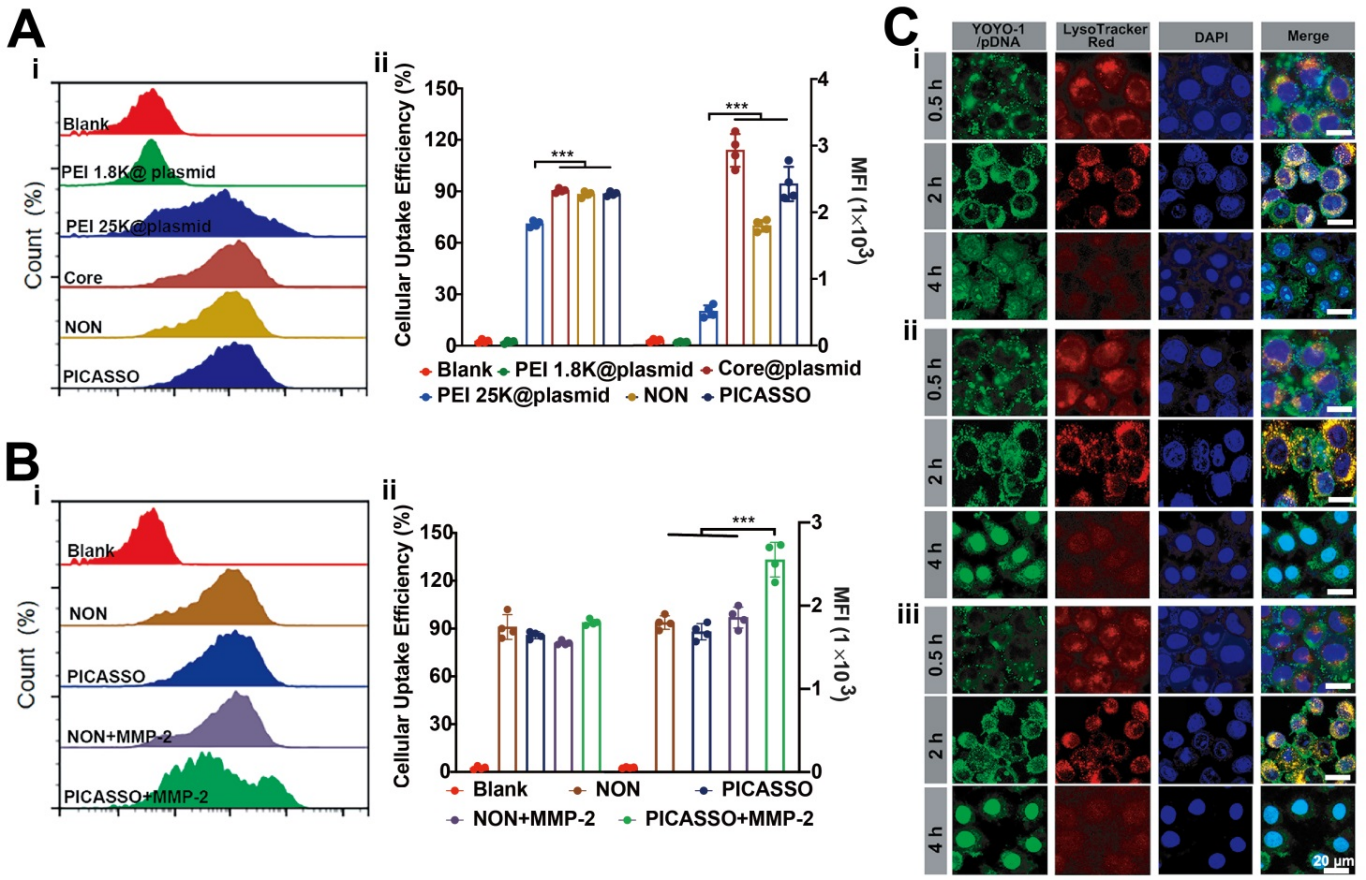
** Cellular internalization behavior.** (**A**) Cellular uptake analysis treated with PICASSO or (**B**) PICASSO + MMP-2. (**C**) Confocal laser scanning microscopy of the lysosomal escape of PEI 25K (i) and PICASSO (ii) or PICASSO + MMP-2 (iii) at 0.5, 2 and 4 h (CRISPRa plasmids were labeled with YOYO-1). Lysosomes were stained with LysoTracker Red and nuclei were stained with Hoechst 33342 (blue). * p < 0.05, and *** p < 0.001.

Subsequently, we evaluated the internalization capability of different formulations *in vitro*. The plasmid was labelled with YOYO-1 (green) for flow cytometric analysis. Because of the integration of HA backbone could improve the cellular uptake of CD44 overexpressing cells 35, thus the CD44 expression of HeLa cells was detected (~100%) (Figure S3). As is shown in Figure 2A, HeLa cells incubated with core@plasmid, NON and PICASSO displayed the higher cellular internalization than PEI 1.8K (92%, 90%, or 85% *vs* 5%, p < 0.001). Moreover, the abovementioned three formulations showed excellent performance of cell uptake compared with PEI 25K (92%, 90%, or 85% *vs* 76%, p < 0.001). The mean fluorescence intensity (MFI) was also consistent with the cellular uptake efficiency. As shown, the highest MFI signal of the core@plasmid indicated excellent cell internalizing capacity of the core of PICASSO. Notably, when in the simulated tumor MMP-2 enzyme condition, the MFI of PICASSO treated group increased significantly, which was the highest in all the groups (Figure 2B). As demonstrated in the results, the MMP-2 responsiveness integrated into the multifunctional shell could significantly enhance the cellular uptake effect of PICASSO.

The capability of endosomal escape plays a key role for efficient delivery of gene drugs which avoided the degradation of plasmid in lysosomes, thus we performed the intracellular trafficking studies (Figure 2C). PEI 25K was chosen as positive control. The PICASSO was labelled with Alexa Fluor-488 and was observed by laser confocal microscopy (LSCM). As shown, the green fluorescence signals of PICASSO were completely merged with the red fluorescence of lysosome (red) after incubating with cells for 2 h, indicating the faster entrance of PICASSO in cells compared with PEI 25K. After 4 h incubation, the ~90% PICASSO escaped successfully from lysosome, and then located with the nucleus smoothly. Meanwhile, the subcellular distribution of PICASSO pre-treated with MMP-2 was also captured by LSCM. We observed that the part of PICASSO pre-treated with MMP-2 had begun to enter the nucleus at 2 h. Moreover, 100% pretreated PICASSO was released into nucleus at 4 h which was much faster than the MMP-2 untreated group. The results demonstrated the rapid process of PICASSO uptake and lysosomal escape in the simulated MMP-2-riched tumor microenvironment.

### Transfection efficiency *in vitro*

**Figure 3:**
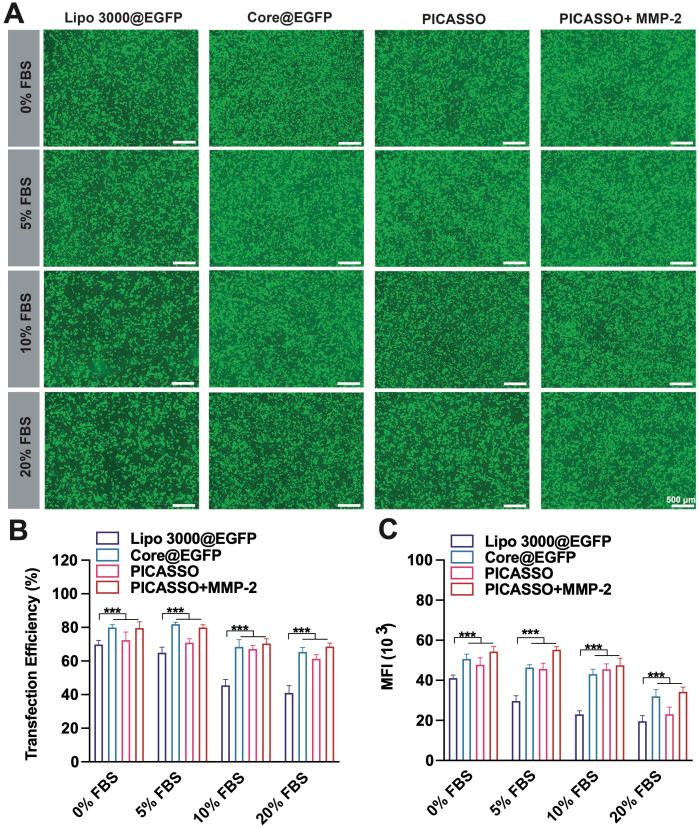
** Transfection efficiency in culture media with 0%~20% serum.** (**A**) fluorescence microscopy images after treated different formulations (scale bar: 500 μm). (**B**) quantitative analysis of GFP^+^ cells (%) by flow cytometry analysis. (**C**) Quantification of the transfection efficiency and MFI. *** p < 0.001.

The intracellular expression of plasmid is the fundamental step to achieve its function. Hence, we evaluated the transfection efficiency of PICASSO in HeLa cells (Figure S4). Plasmid expressing enhanced green fluorescent protein (pEGFP) with the similar size (~10 kb) as CRISPRa plasmid was used as the reporter gene. As the results, PEI 1.8K showed an extremely low transfection efficiency of HeLa cells (4%). Although the transfection rate of PEI 25K was 48%, the efficiency is not potent enough to realize CRISPRa function. In contrast to PEI 25K, PICASSO, NON and core@plasmid showed much higher transfection efficiency of 78%, 76% and 82% respectively. Furthermore, the MFI was consistent with transfection efficiency. More importantly, the ability to resist blood interference is the essential property for clinical application of the CRISPRa system *in vivo.* Subsequently, we assessed the transfection stability of PICASSO in serum with various concentration gradients (0~20%, Figure 3A). Lipofectamine 3000, the commercial reagent with high transfection performance, was used as a control. As shown, the interference of serum only has a minor impact on the transfection efficiency of PICASSO in the MMP-2 condition (from ~76% to ~73%) when confront the introduction of serum with various concentrations, even the serum concentration up to 20%. In contrast, the transfection rate of Lipofectamine 3000 group was reduced dramatically (from ~68% to ~40%) (p < 0.001) under the same condition (Figure 3B). Similarly, the PICASSO in the simulated tumor condition indicated the highest MFI among all the groups due to the responsiveness of MMP-2 enzyme (Figure 3C, Figure S5). The results indicated the transfection capability of PICASSO could remain high efficiency and stability under the condition of serum interference.

### Activation of endogenous gene targets in cancer cells

**Figure 4:**
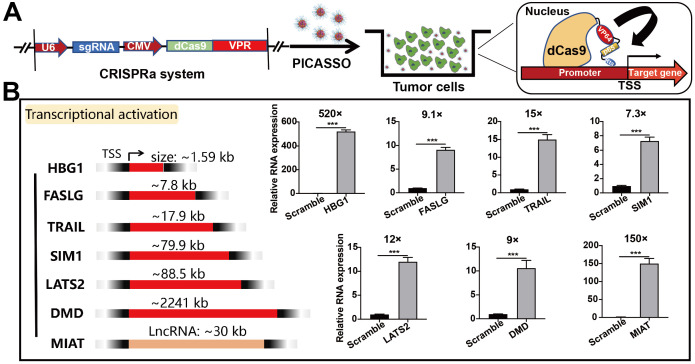
** Activation of endogenous gene targets in HeLa cells.** (**A**) Schematic diagram of CRISPRa plasmid system. (**B**) Quantitative real-time PCR analysis of various targets containing genes ranging in size from ~1 kb to ~2000 kb and LncRNA. *** p < 0.001.

Having performed initial characterization, we next investigated the capability of PICASSO to activate endogenous transcription with various gene types (coding and noncoding targets) (Figure 4A). To this end, we selected a series of gene targets associated with cellular reprogramming (HBG1, 1.59 kb), ontogeny (SIM1, 79.9kb; DMD, 2241 kb) and oncological treatment (FASLG, 7.8 kb; TRAIL, 17.9 kb; LATS2, 88.5kb) for the subsequent evaluation. PICASSO showed great transcriptional activation levels across the panel of endogenous targets respectively (HBG1, 520-fold; FASLG, 9.1-fold; TRAIL, 15-fold; SIM1, 7.3-fold; LATS2, 12-fold; DMD, 9-fold; MIAT, 150-fold) (Figure 4B). Therein, DMD is a potential therapeutic target for Duchenne muscular dystrophy 37. But targeting DMD has insurmountable challenges: the gene is too large to be contained in the carriers that are traditionally used to deliver genetic cargos 38. Notably, the activated expression of DMD can be achieved by utilizing the PICASSO. Even for the long noncoding RNA such as MIAT1, the nanocomplex can still reach 150-fold expression activation. In addition, we also noted a difference of gene activation levels between diverse targets which was consistent with previous studies (genes with high basal expression were less potently activated) 13. As it turns out, the robust gene activation ability of PICASSO surmounts the limitation of various gene types and size, provides a powerful means for gene therapy.

### Anti-tumor efficacy *in vitro*

**Figure 5:**
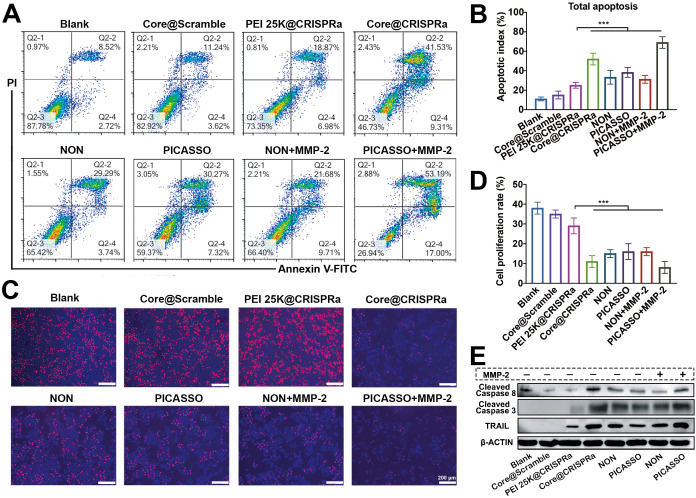
** Anti-Tumor efficacy *in vitro*. (A**, **B**) Apoptosis of HeLa cells after different compound treatments evaluated by flow cytometry. (**C**, **D**) Inhibitory effects of various treatments on cell proliferation detected by EdU analysis. (**E**) Western blot evaluation of HeLa cells through different treatments. *** p < 0.001.

Considering the safety and effectiveness of tumor treatment, the ability to selectively induce apoptosis of tumor cells but not normal cells make the tumor necrosis factor-related apoptosis-inducing ligand (TRAIL) an ideal therapeutic target among the abovementioned results 39,40. We thus selected TRAIL gene as the model target for cancer therapy and proceeded to evaluate the anti-tumor efficiency of the PICASSO *in vitro*. After 48 h treatment, the cell apoptosis assays were conducted (Figure 5A-B). There was no significant difference in the apoptosis ratio between Core@scramble (scramble: the CRISPRa plasmid containing the nonsence sgRNA sequence) and blank groups. When target TRAIL locus, the PICASSO and NON induced higher apoptosis rates than that of PEI 25K (~38% or ~33% *vs* ~25%, p < 0.001). Notably, the vast majority of tumor cells (~70%) were induced apoptosis in the PICASSO group when confronts the MMP-2 enzyme condition. Moreover, the cell proliferation was measured by an EdU assay (Figure 5C-D). The PICASSO group exhibited 93% decrease in the proliferation rate of HeLa cells in the MMP-2 condition which was the highest in all groups. Meanwhile, the protein levels of TRAIL were further confirmed after different formulations treatments, suggesting that the CRISPRa system was successfully delivered into tumor cells and dramatically upregulated the protein expression of TRAIL by PICASSO mediated transcription activation (Figure 5E, Figure S6). These results demonstrated the powerful anti-tumor effect of PICASSO *in vitro*.

### Tumor targeting capability and antitumor efficacy *in vivo*

**Figure 6:**
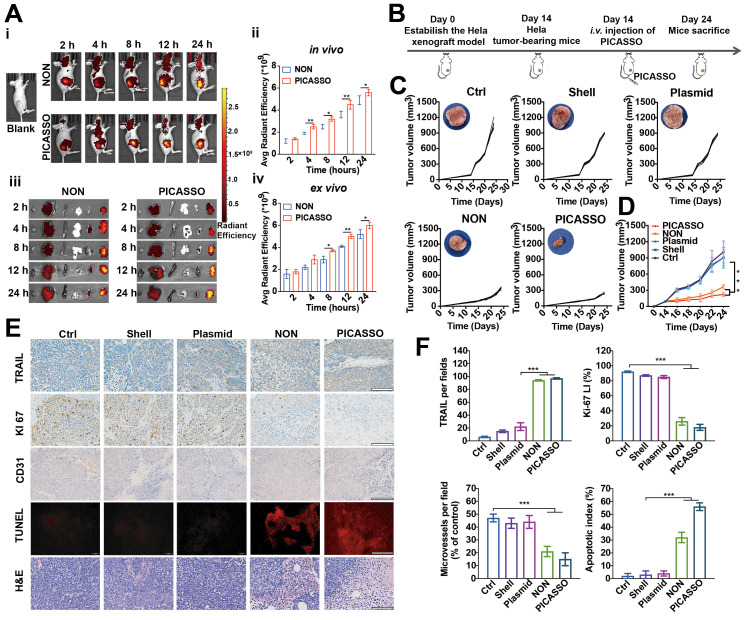
** Biodistribution and anti-tumor efficacy of PICASSO *in vivo*.** (**A**) Fluorescence photographs and quantitative intensity of HeLa xenograft tumor models after intravenous administration of different groups (*ex vivo* images from left to right: heart, liver, spleen, lung, kidney, and tumor). (**B**) Schematic diagram of *in vivo* treatment of HeLa xenograft tumor models. (**C**) Individual tumor growth profiles and average tumor volume (**D**) of mice treated with various agents. (**E**) IHC analyses of the expression of TRAIL, KI67, and CD31 in tumor sections from different groups as well as H&E and TUNEL staining (scale bar: 50 μm). (**F**) Mean TRAIL, mean Ki67 LI, microvessels density and apoptosis index in each treatment group. * p < 0.05, ** p < 0.01 and *** p < 0.001.

Moreover, to explore the tumor targeting capability, the distribution profiles of TOTO-3-labeled PICASSO in tumor xenograft models were collected (Figure 6B). After 2 h intravenous injections, obvious fluorescence signals were observed in the tumor region and increased up to a peak at 24 h, indicating the excellent tumor targeting capacity of the PICASSO and NON. In particular, the fluorescence signal of PICASSO group was stronger than that of NON group from 4 h. Meanwhile, the tumors and vitals of the tumor-bearing mice were harvested for further quantification of the fluorescence intensity. In contrast to the NON group, higher fluorescence signals of the PICASSO group in xenograft models demonstrated once again that the programmed hierarchical-responsive property could enhance tumor accumulation and duration of the nanocomplex *in vivo*, which was crucial to the enhanced therapeutic performance and minimum adverse effects of CRISPRa system.

Inspired by the anti-tumor efficacy *in vitro* and tumor targeting capability, the *in vivo* therapeutic effect of the PICASSO was next investigated in tumor bearing mice. The therapeutic profiles utilized *in vivo* were shown in Figure 6A. Tumors treated with PBS, Shell (the shell of PICASSO) or the naked plasmid of CRISPRa-TRAIL showed rapid growth. In contrast, the PICASSO and NON groups showed efficient inhibition of tumor growth. Notably, the tumor growth in the PICASSO group exhibited prompt tumor regression, a better result than that of NON formulation treatment which was further confirmed by the measurement of tumor weight (Figure 6C-D, Figure S7A). The data showed that PICASSO group had the minimum tumor weight (~0.1 g) than other treatments. In addition, no obvious influence in the body weight was observed during the observation period per group, indicating a good biocompatibility of the nanocomplexes (Figure S7B). Together, these results validated that PICASSO could enhance the CRISPRa-mediated therapeutic effect which benefit from the MMP-2 responsiveness triggered deep penetration and augment the cellular uptake ability in the tumor microenvironment.

In the following experiments, the tumor tissues from each group were harvested for the hematoxylin-eosin (H&E) staining, immune-histochemical assay (IHC) and dUTP nick end-labeling (TUNEL) analysis (Figure 6E-F). The data further supported that PICASSO activated the protein level of TRAIL (~86%) and suppressed tumor cell proliferation (Ki67 staining) (~15%) significantly compared with other groups. Afterwards, the tumor treated with PICASSO showed more apoptotic cells (~46%) than other treatments verified by the TUNEL analysis. On the other hand, the microvessel density confirmed by CD31 staining dramatically diminished in the PICASSO treatment group (~8%), and thus confirmed the former result that the PICASSO has the best anti-tumor efficacy among all the treatment groups.

In addition, the potential toxicity of PICASSO was analyzed by H&E. No lesions in heart, liver, spleen, lung and kidney were observed after the treatment of the investigated formulations for 4 weeks (Figure S8). The results also indicated that the treatment decreased immunocyte infiltration into the major organs. Taken together, the PICASSO platform enabled effective suppression of tumor growth via CRISPRa-mediated robust transcriptional activation without genome editing.

## Conclusion

In summary, PICASSO were successfully constructed for the CRISPRa-mediated transcriptional activation of various endogenous gene types and the following tumor treatment. The design of the programmed hierarchical-responsive structure not only endow PICASSO with circulating stability and accurate tumor accumulation, but also facilitated cell entry and endosomal escape of CRISPRa payload when facing the environment inside and outside tumor cells. Benefit from the integrity of the function and structure, the PICASSO realized robust activation of various gene types containing genes ranging in size from ~1 kb to ~2000 kb and LncRNA. More importantly, *in vivo* results suggested that by CRISPRa mediated efficient transcriptional activation of the therapeutic target, PICASSO dramatically inhibited tumor proliferation and reduced the tumor burden with a favorable safety profile in xenograft tumor models. Taken all together, the proposed strategy pioneers a new avenue for CRISPRa-based next generation cancer therapy, breaking the restrictions on gene size and type and broaden the therapeutic promise for numerous diseases.

## Supplementary Material

Supplementary figures and table.

## Abbreviations

CRISPRa: CRISPR activation system
LncRNA: long noncoding RNA
PICASSO: Programmable hierarchical-responsive nanoCRISPR
NON: MMP-2-nonresponsive nanoparticles
LHRH: luteinizing hormone-releasing hormone
HA: hyaluronic acid
MMP-2: matrix metalloproteinases-2
PHE: phenylalanine
TYR: tyrosine
^1^*H* NMR: ^1^*H* nuclear magnetic resonance analysis
DLS: Dynamic light scattering
TEM: transmission electron microscopic
PEI 25K: Branched polyethyleneimine 25 kDa
PEI 1.8K: polyethyleneimine 1.8 kDa
MFI: mean fluorescence intensity
LSCM: laser confocal microscopy
TRAIL: tumor necrosis factor-related apoptosis-inducing ligand
H&E: hematoxylin-eosin staining
IHC: immune-histochemical assay
TUNEL: TdT-mediated
dUTP: nick end labeling
IHC: Immunohistochemistry
