# Supplementary material for: A programmable hierarchical-responsive nanoCRISPR elicits robust activation of endogenous target to treat cancer
Source: Theranostics. 2021 Oct 11;11(20):9833–46. doi: 10.7150/thno.62449 (PMC8581410; doi:10.7150/thno.62449)
Supplement: Supplementary file 1 — Supplementary figures and table. [file thnov11p9833s1.pdf]

Supplementary Material

A programmable hierarchical-responsive nanoCRISPR elicits robust activation of endogenous target to treat cancer

Chao Liu [#], *Ning Wang* [#], *Rui Luo, Lu Li, Wen Yang, Xiye Wang, Meiling Shen, Qinjie Wu* [*], *Changyang Gong* [*]

State Key Laboratory of Biotherapy and Cancer Center, West China Hospital Sichuan University, Chengdu, 610041, P. R. China

[*] To whom correspondence should be addressed (C Gong and Q Wu). E-mail: chygong14@163.com and cellwqj@163.com.

[#] These authors contributed equally to this work.

Table S1. The sequence information of sgRNAs and primers.

Name	Sequence information
TRAIL-F	GAGCTGAAGCAGATGCAGGAC
TRAIL-R	TGACGGAGTTGCCACTTGACT
HBG1-F	AATGTGGAAGATGCTGGAGG
HBG1-R	GCCAAAGCTGTCAAAGAACC
SIM1-F	CGCGGACTAGGAGGGAGAA
SIM1-R	GTCGTGAGTCTGATTATGGATGC
DMD-F	CAACTTATTGGCATGATGGAGTG
DMD-R	GTAGGTCACTGAAGAGGTTCTC
MIAT-F	TGGCTGGGGTTTGAACCTTT
MIAT-R	AGGAAGCTGTTCCAGACTGC
FASLG-F	AAAGGAGCTGAGGAAAGTGG
FASLG-R	CATAGGTGTCTTCCCATTCCAG
LATS2-F	AACTCACAGATTTCGGCCTC
LATS2-R	ACACCGACAGTTAGACACATC
ACTB-F	CGGGAAATCGTGCGTGACATTAAG
ACTB-R	TGATCTCCTTCTGCATCCTGTCGG
TRAIL loci	AGAAGAGAGAAATGGGCTTG
HBG1 loci	TCCCTGAACTTTTCAAAAAT
SIM1 loci	AGACTGAAGAGCAAACGTCA
DMD loci	ACTCTACTATTGTTACACTT
MIAT loci	GCGCCCATGAAATTTTAATG
FASLG loci	CATAGCCTACTAACCTGTTT
LATS2 loci	CAAATAGGTCCGAGAGGCCC

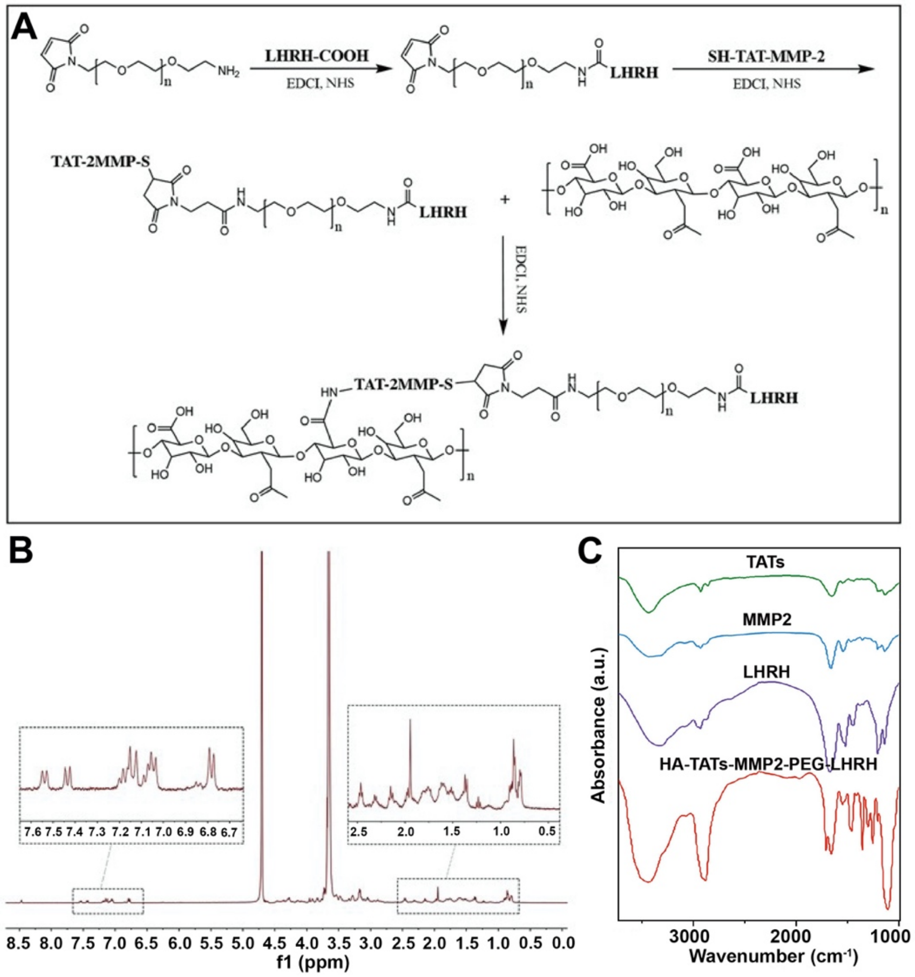

Figure S1. The synthetic route (A), [1]*H* NMR (B) and FTIR spectra (C) of the shell.

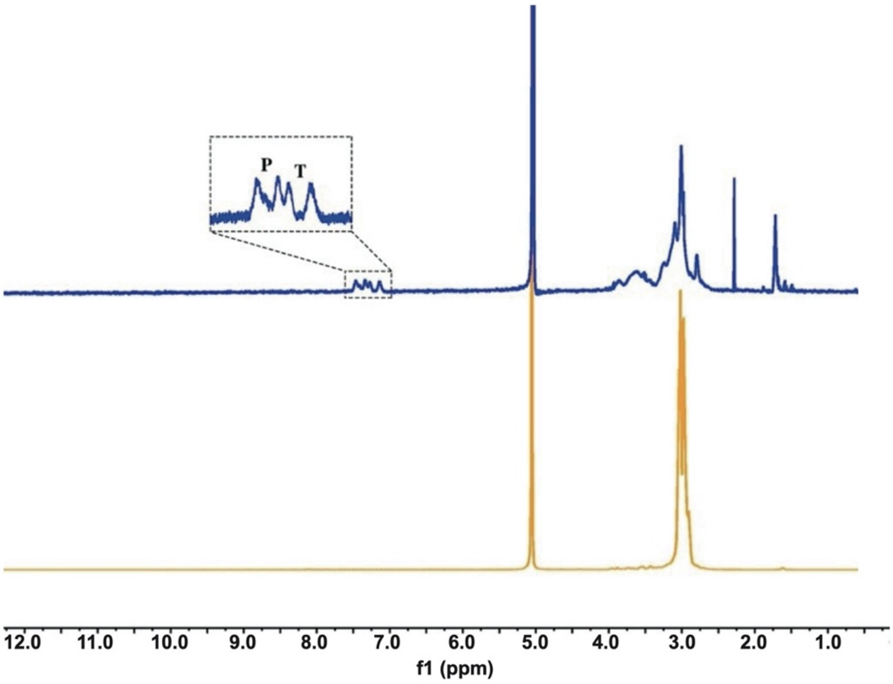

Figure S2. ^{1}H NMR spectra of the core.

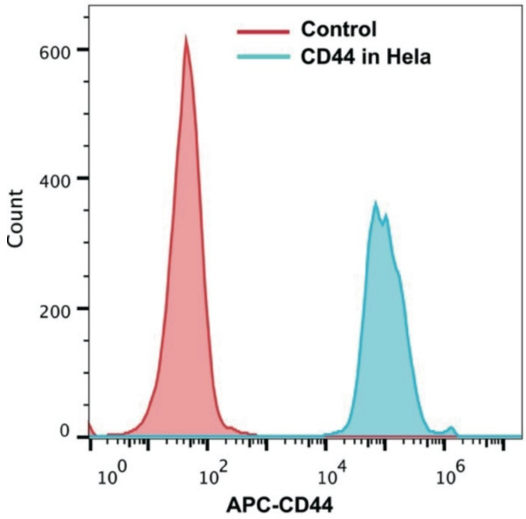

Figure S3. Investigation of the expression of CD44 receptor on surface of HeLa cells by flow cytometry.

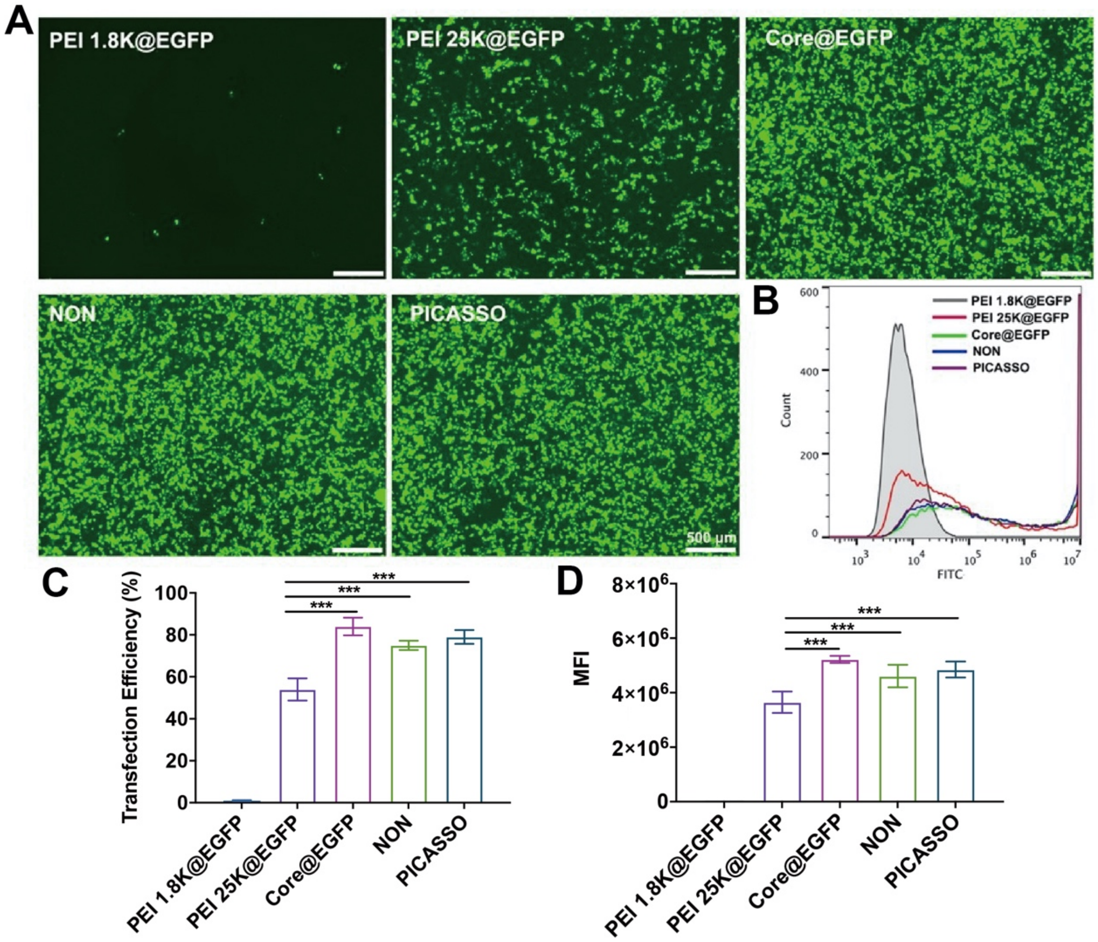

Figure S4. Evaluation of the transfection efficiency of different compounds. (**A**) Fluorescent photographs of HeLa cells after transfected for 24 h. (**B-D**) Quantitative analysis by flow cytometry analysis.

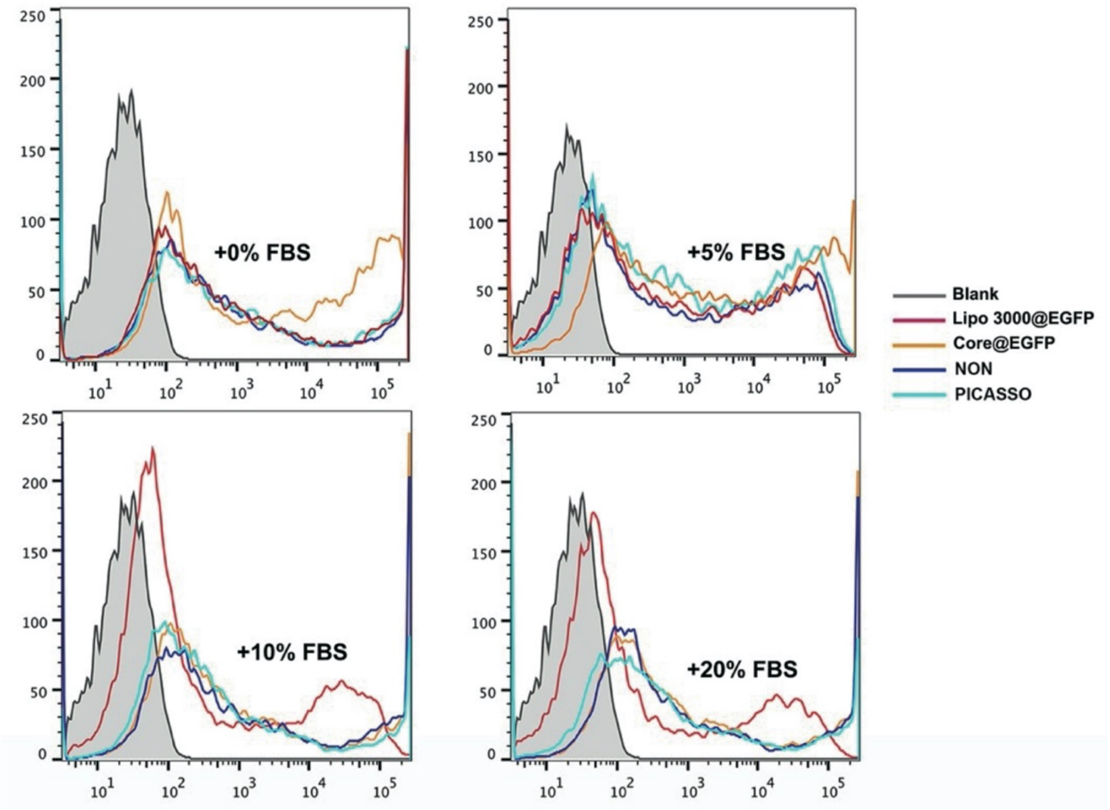

Figure S5. Quantitative analysis of transfection efficiency under 0~20% FBS medium.

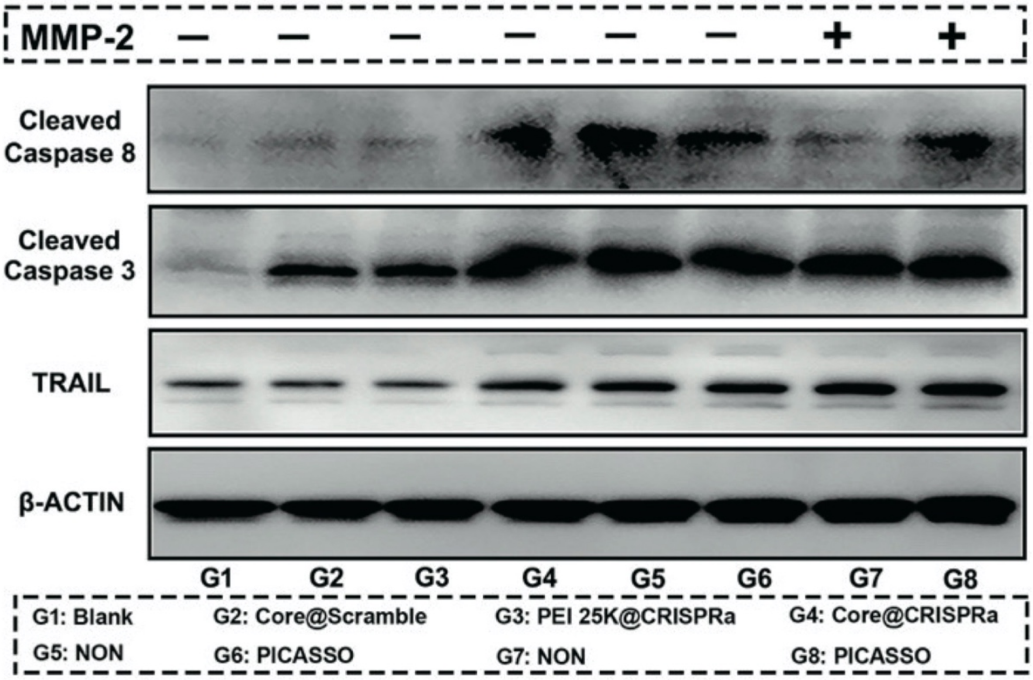

Figure S6. The repeated experiment of western blot.

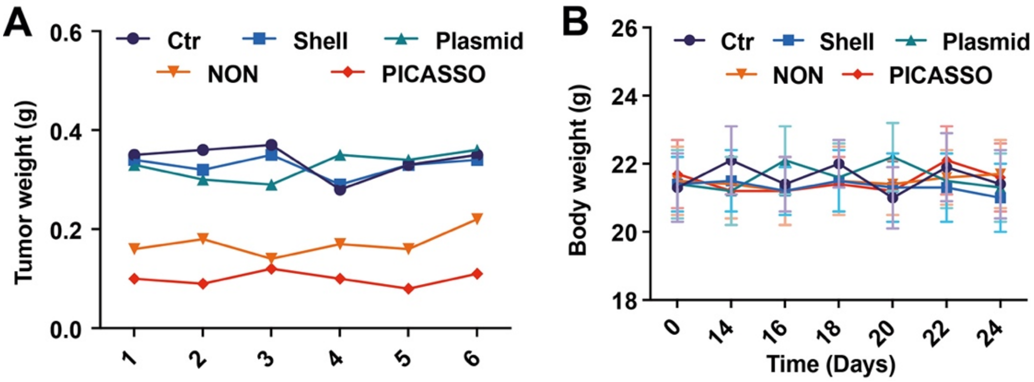

Figure S7. (**A**) Tumor weight and (**B**) body weight of mice after different treatments.

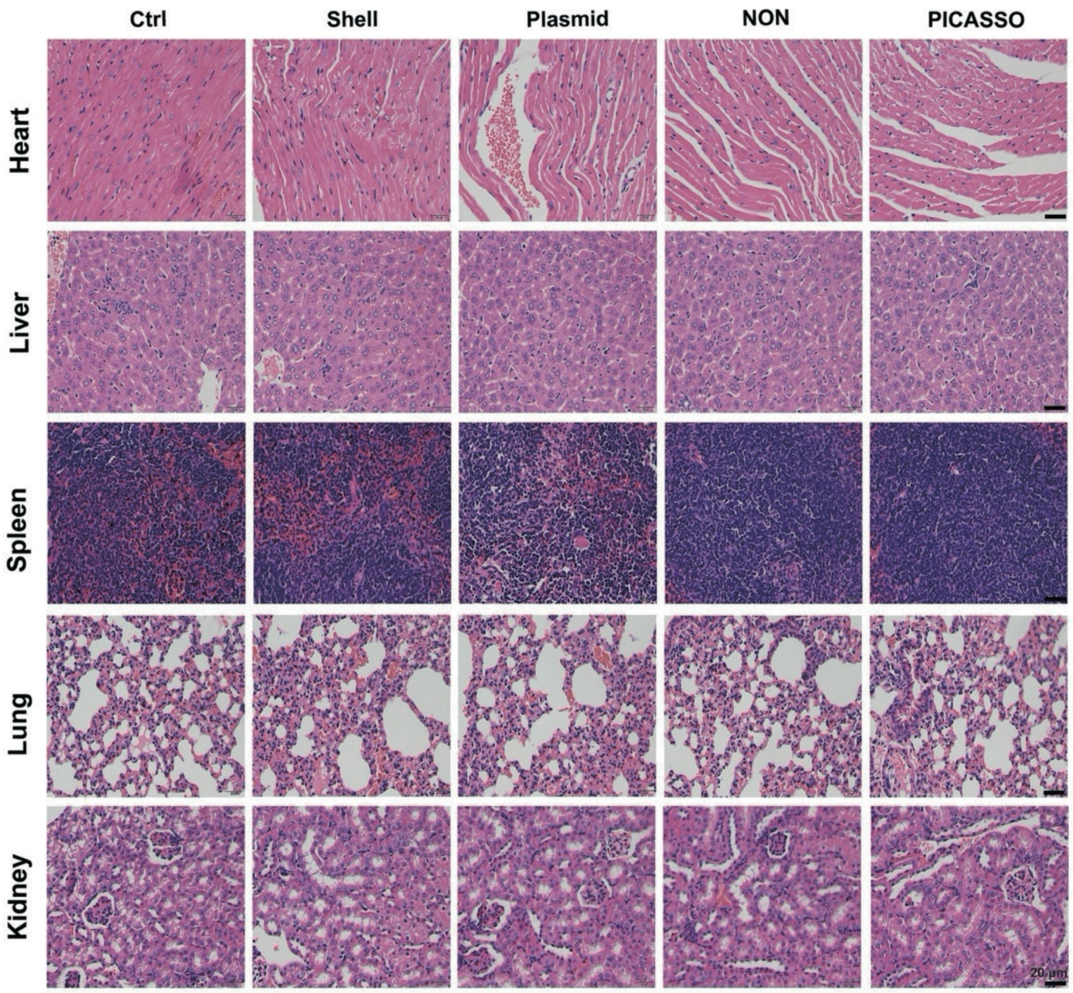

Figure S8. H&E staining of main organs for toxity analysis.
